# Microbial regulation of intestinal motility provides resistance against helminth infection

**DOI:** 10.1038/s41385-022-00498-8

**Published:** 2022-03-14

**Authors:** Mati Moyat, Luc Lebon, Olaf Perdijk, Lakshanie C. Wickramasinghe, Mario M. Zaiss, Ilaria Mosconi, Beatrice Volpe, Nadine Guenat, Kathleen Shah, Gillian Coakley, Tiffany Bouchery, Nicola L. Harris

**Affiliations:** 1 Global Health Institute, Swiss Federal Institute of Technology (EPFL), Lausanne, 1015 Lausanne, Switzerland; 2 Department of Immunology and Pathology, Central Clinical School, Monash University, The Alfred Centre, Melbourne, VIC Australia; 3 Department of Internal Medicine 3, Rheumatology and Immunology, Friedrich-Alexander-University Erlangen-Nürnberg (FAU) and Universitätsklinikum Erlangen, Erlangen, Germany

## Abstract

Soil-transmitted helminths cause widespread disease, infecting ~1.5 billion people living within poverty-stricken regions of tropical and subtropical countries. As adult worms inhabit the intestine alongside bacterial communities, we determined whether the bacterial microbiota impacted on host resistance against intestinal helminth infection. We infected germ-free, antibiotic-treated and specific pathogen-free mice, with the intestinal helminth *Heligmosomoides polygyrus bakeri*. Mice harboured increased parasite numbers in the absence of a bacterial microbiota, despite mounting a robust helminth-induced type 2 immune response. Alterations to parasite behaviour could already be observed at early time points following infection, including more proximal distribution of infective larvae along the intestinal tract and increased migration in a Baermann assay. Mice lacking a complex bacterial microbiota exhibited reduced levels of intestinal acetylcholine, a major excitatory intestinal neurotransmitter that promotes intestinal transit by activating muscarinic receptors. Both intestinal motility and host resistance against larval infection were restored by treatment with the muscarinic agonist bethanechol. These data provide evidence that a complex bacterial microbiota provides the host with resistance against intestinal helminths via its ability to regulate intestinal motility.

## Introduction

The large majority of mammals harbour parasitic helminths within their intestine, with individual worms often able to live for several years within a single host^1^. Although these parasites have been largely eradicated within westernised human populations, the World Health Organization reports that ~1.5 billion people continue to suffer from infection with intestinal helminths across the world^2^. Helminths have exerted a strong force on mammalian evolution and are thought to represent the main force underlying the selection of human genes associated with autoimmunity^3^. The protective immune response that evolved against helminths has largely been characterised in mice and is dominated by a strong type 2 immune response that includes the activation of type two innate lymphoid cells (ILC2) by IL-33, IL-25 and neuromedin U released from necrotic epithelial cells, tuft cells, or sensory enteric neurons respectively^4^. This is followed by the activation of type 2 CD4^+^ T cells (Th2)^5^ and production of both polyclonal and parasite-specific IgG and IgE^6,7^. Following primary infection, the type 2 cytokines IL-4 and IL-13, play a major role in the expulsion of adult worms by activating non-immune cells to promote intestinal secretions and muscle contractility, culminating in what is commonly referred to as the “weep and sweep” response^8^. This response is thought to mediate the active expulsion of worms by increasing mechanical forces in addition to providing an inhospitable environment to the parasite through the secretion of goblet cell-derived mucins^9^ and resistin-like molecule β (RELM-β)^10^. Soil-transmitted helminths reside in intestine alongside a complex bacterial community with the microbiota^1^. The intestinal microbiota can impact on diverse processes such as digestion, metabolism and post-natal immune development and function^11^. It also acts to limit host susceptibility to pathogens, a property commonly known as “colonisation resistance”^12,13^. Increasing evidence indicates that helminths alter the complex bacterial communities present within the intestine^14^, and that helminth-bacterial interactions result in alterations to the host immune responsiveness and can provide protection against inflammatory disorders^15,16^. Helminth infection can also alter host susceptibility to pathogen infection and a recent report demonstrates that the presence of an intestinal helminth at the time of *Salmonella* infection aids the establishment of *Salmonella* within the small intestinal lumen^17^. These findings indicate that disruption of one member of the intestinal ecosystem can have important implications on other members and/or on the host immune system.

In this study we set out to investigate whether the presence or absence of a complex bacterial microbiota impacts on host resistance against intestinal helminth infection. For this purpose we used the natural murine helminth parasite, *Heligmosomoides polygyrus bakeri* (*Hpb*), which establishes chronic infections and has a purely enteric lifecycle similar to the common trichostrongylid infections of domestic ruminants^6^. Although *Hpb* secretes immuno-modulatory molecules that allow it to establish chronic infections in susceptible mouse strains, immuno-competent mice eventually expel the adult worms and are resistant to repeated infection^18^. In the current study we observed increased parasite numbers in mice lacking a complex bacterial microbiota. Surprisingly, the same mice exhibited a normal or enhanced type 2 immune responses. We therefore investigated a possible contribution of intestinal physiology to host resistance against the parasite and identified an important contribution of microbial-induced increases in intestinal motility. Together, these observations advance our understanding of the complex interactions between intestinal helminths and the intestinal microbiota by revealing a role for the microbiota in providing host resistance against intestinal helminths.

## Results

### The microbiota provides host resistance to intestinal helminth infection

*Hpb* infection of mice can be established following oral gavage of infective stage-three larvae (L3). By 24 h post-infection, the larvae penetrate the wall of the upper small intestine and undergo several moult cycles and mature into stage-four larvae (L4) by day 3–4 post-infection. By day 8–9 post-infection they become adult worms and exit the tissue to reside within the intestinal lumen. Fecund adult worms can persist within their host for several months before being expelled by the host^19,20^. To determine the impact of the bacterial microbiota on *Hpb* infection we used two intestinal models established in the laboratory for microbiome-helminth research. In the first model, antibiotic treatment was used to deplete the intestinal microbiota both before and during *Hpb* infection. In the second model germ-free mice, for which the microbiota is absent from birth, were infected with *Hpb*. For all experiments, mice were infected with specially hatched axenic *Hpb* larvae using a previously reported protocol^16,21^. The same batch of larvae was also used to infect specific pathogen-free (SPF) mice, as the age-matched positive control group. Antibiotics treatment was effective in depleting most bacteria as shown by a significant reduction in bacterial load (Fig. S1A) and diversity in the caecum (Fig. S1B). Moreover,16S rRNA amplicon sequencing revealed that antibiotic treatment altered the bacterial composition in the caecum (Fig. S1C) and ileum (Fig. S1E) with *Muribaculaceae*, representing the main bacterial family to persist at both sites following antibiotic treatment (Fig. S1D, F).

**Fig. 1:**
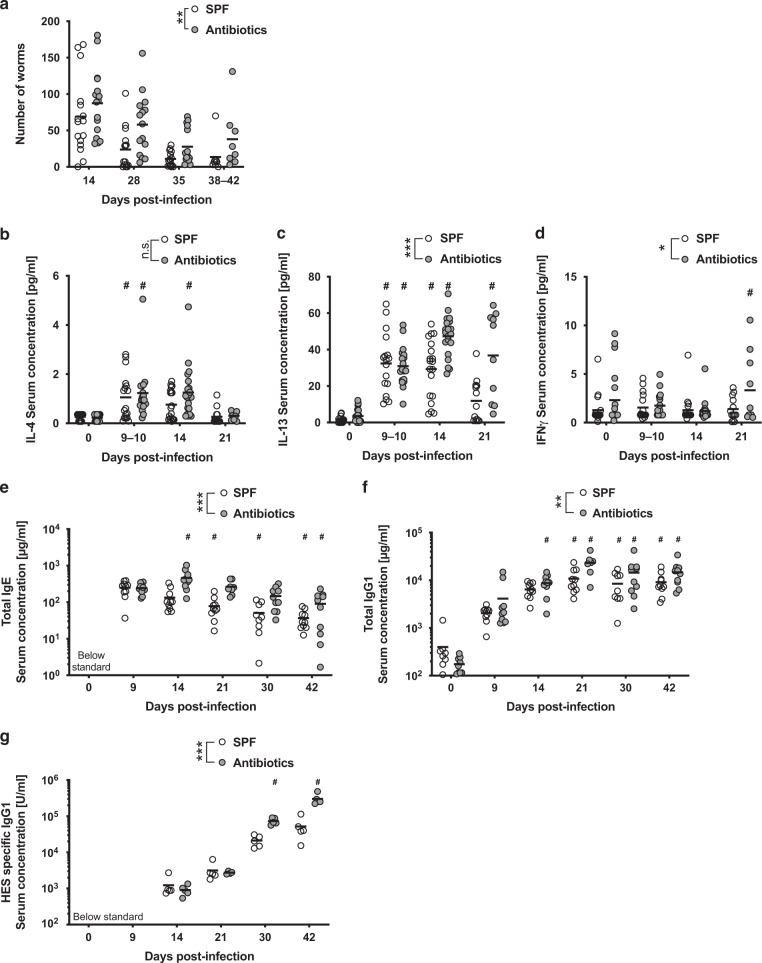
*Hpb* numbers are increased in microbiota-depleted animals despite evidence of a strong type 2 immune response. **a** SPF and antibiotic-treated mice (enrofloxacin for 2 weeks then amoxicillin and clavulanic acid for the rest of the experiment) were infected for 14 to 42 days with 200 *Hpb* larvae. **a** Number of adult worms in the intestine were assessed at each time points. Data are pooled from four independent experiments and analysed using two-way ANOVA. The number of mice per time point are: D14 = 16, D28 = 15, D35 = 16 and D38-42 = 8 for each group. **b**–**d** SPF and antibiotic-treated mice (enrofloxacin for 2 weeks then amoxicillin and clavulanic acid for the rest of the experiment) were infected for 0 to 21 days with 200 *Hpb* larvae. Concentration of IL-4 (**b**), IL-13 (**c**) and IFNγ (**d**) were measured by ELISA. Data were pooled from four independent experiments and analysed using two-way ANOVA. The number of mice per time point are: (1) for SPF mice D0 = 19, D9-10 = 17, D14 = 19 and D21 = 13; (2) for antibiotic-treated mice D0 = 22, D9-10 = 18, D14 = 20 and D21 = 10. **e**–**g** SPF and antibiotic-treated mice (enrofloxacin for 2 weeks then amoxicillin and clavulanic acid for the rest of the experiment) were infected for 0 to 42 days with 200 *Hpb* larvae. Concentration of total circulating IgE (**e**), IgG1 (**f**) and HES specific IgG1 (**g**) were measured using ELISA. Data are pooled from two independent experiments and analysed using two-way ANOVA. **e**, **f** The number of mice per time point are: (1) For SPF mice D0 = 8, D9 = 10, D14 = 10, D21 = 10, D30 = 9 and D42 = 10; (2) for antibiotic-treated mice D0 = 9, D9 = 10, D14 = 10, D21 = 7, D30 = 10 and D42 = 10. **g** The number of mice per time point are: (1) For SPF mice D0 = 5, D9 = 5, D14 = 5, D21 = 5, D30 = 5 and D42 = 5; (2) for antibiotic-treated mice D0 = 5, D9 = 5, D14 = 5, D21 = 4, D30 = 5 and D42 = 5. *display statically significant differences between groups and # display statistically significant differences compared to the naïve group (0 days post-infection).

As expected, adult worm numbers in SPF mice peaked between weeks 2 and 4 following infection and diminished thereafter, with very few worms persisting after week 6 (Figs. 1a and S2A). In comparison, infection of antibiotic-treated (Fig. 1a) or germ-free (Fig. S2A) mice resulted in significantly increased numbers of adult worms across all investigated time points. Infected antibiotic-treated mice also exhibited increased egg production compared to SPF mice (Fig. S1B, C). The finding that *Hpb* can complete its lifecycle in the absence of a complex microbiota suggests that it likely feeds on host tissue rather than bacteria within the host, supporting previous research^22^. These findings also indicate that the presence of a complex microbiota favours the host, allowing increased resistance against the parasite.

### Microbiota depleted mice mount robust type 2 immune responses following intestinal helminth infection

Given the known role of type 2 immunity in promoting expulsion of adult worms from the intestinal lumen^20^, we next set out to investigate whether the cytokines and antibodies associated with type 2 immune cells were altered in microbial depleted mice. *Hpb* infection elicited a significant increase in type 2 cytokines; IL-4 and IL-13 in the serum (Fig. 1b, c) and peritoneum (Fig. S2D, E) in both SPF and antibiotic-treated mice. Interestingly, serum IL-13 was increased in antibiotic-treated compared to SPF mice (Fig. 1c), whilst serum IL-4 (Fig. 1b), and peritoneal IL-4 and IL-13 (Fig. S2D, E), levels were similar between the two groups. Infection also elicited production of the type 1 cytokine, IFNγ, in the peritoneum of SPF but not antibiotic-treated mice. *Hpb* infection did not impact on serum IFNγ (Fig. 1d), however antibiotic-treated mice exhibited higher baseline levels of this cytokine within the serum (Fig. 1d).

We next investigated cytokine production from CD4^+^ T cells harvested from the draining mesenteric lymph nodes (MLNs) then subjected to mitogenic stimulation in vitro. Similar numbers of IL-4 producing and IL-13 producing MLN Th2 cells were elicited by *Hpb* infection in SPF and antibiotic-treated mice (Fig. S2G, H). In line with the peritoneal response, IFNγ producing Th1 cells were increased by *Hpb* infection, but this increase was less dramatic in antibiotic-treated-mice as compared to SPF mice (Fig. S2I). *Hpb* induced production of increased levels of IFNγ in SPF compared to antibiotic-treated mice likely results from parasite-induced damage to the intestinal epithelium allowing bacterial translocation from the lumen into the underlying tissues. This was further supported by observations of a transient increase in FITC-dextran leakage from the intestinal lumen into the blood stream of infected SPF mice (Fig. S2J), which occurred around the same time as adult worms migrate from the intestinal wall back into the intestinal lumen (day 7, Fig. S2J).

**Fig. 2:**
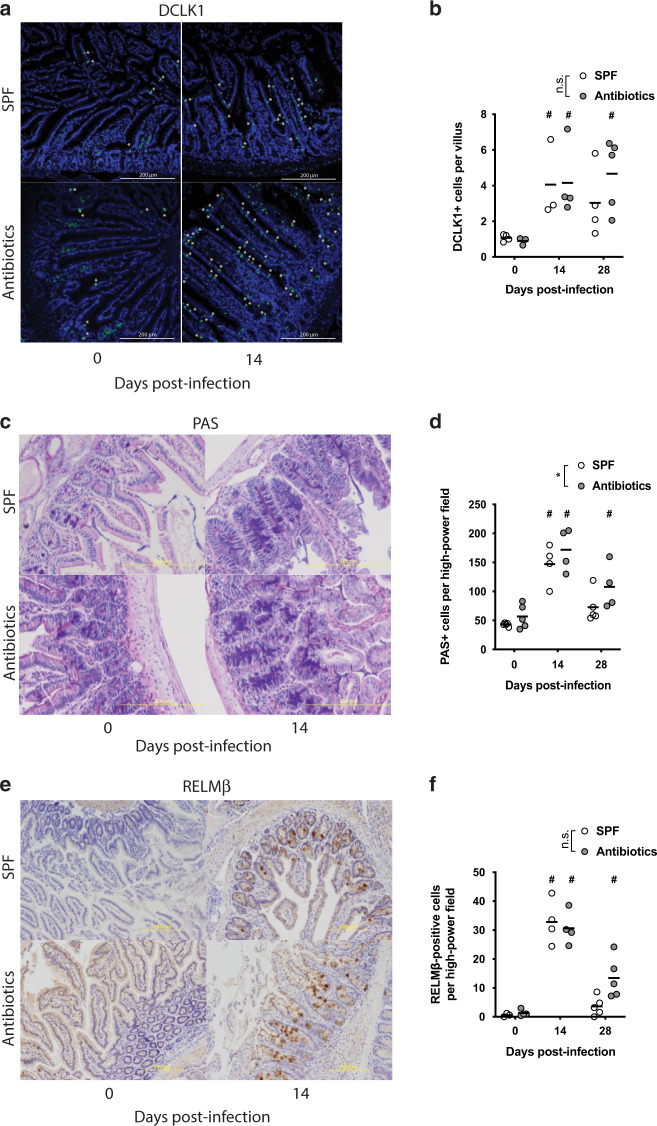
Antibiotic treatment does not impair the expansion of type 2 immune-dependent intestinal epithelial cell subtypes. **a**–**f** SPF and antibiotic-treated mice (enrofloxacin for 2 weeks then amoxicillin and clavulanic acid for the rest of the experiment) were infected for 0 to 21 days with 200 *Hpb* larvae. Intestine were processed for histological staining of (**a**) DCLK1 (tuft cells (*), green staining) (**c**) mucus with PAS (goblet cells, purple staining) or (**e**) RELM-β (goblet cells, brown staining). All scales represent 200 μm. For each staining, representative images of day 0 and 14 post infected are displayed. Quantification of (**b**) DCLK1^+^, (**d**) mucus-producing and (**f**) RELM-β^+^ cells are represented as an average of at least five fields of view for each individual. Data were pooled from two independent experiments with one experiment including day 0 and 14 time points and the 2nd experiment including the day 28 time point. Each dot represents and individual animal. Data were analysed using two-way ANOVA. **b** The number of mice per time point are: (1) for SPF mice D0 = 4, D14 = 3 and D28 = 4; (2) for antibiotic-treated mice D0 = 3, D14 = 4 and D28 = 5. **d** The number of mice per time point are: (1) for SPF mice D0 = 3, D14 = 4 and D28 = 5; (2) For antibiotic-treated mice D0 = 4, D14 = 4 and D28 = 5. **f** The number of mice per time point are: (1) for SPF mice D0 = 4, D14 = 3 and D28 = 4; (2) for antibiotic-treated mice D0 = 3, D14 = 4 and D28 = 5. * display statically significant differences between groups and # display statistically significant differences compared to the naïve group (0 days post-infection).

*Hpb* also drives a T-cell-dependent polyclonal IgE and IgG1 response which functions to limit worm fitness^6,7^, and a delayed parasite-specific response that contributes to protection against re-infection^7,23^. Serum titres of the IL-4 associated IgE and IgG1 isotypes were markedly increased following *Hpb* infection and were present at higher levels in the antibiotic-treated compared to SPF mice (Fig. 1e, f). A similar trend was observed for parasite-specific IgG1 (Fig. 1g). In support of the robust innate and adaptive type 2 cell responses observed in both SPF and antibiotic-treated mice following *Hpb* infection, we observed similar numbers of tuft cells (Fig. 2a, b) and mucus-producing goblet cells (Fig. 2c, d) at day 14 and day 28 post-infection. Examination of the number of RELM-β positive goblet cells revealed that these cells increased in response to *Hpb* infection with similar numbers observed at day 14 post-infection (Fig. 2e, f). Interestingly, the number of RELM-β positive cells remained elevated above background levels in antibiotic-treated mice at day 28 post-infection, whereas they returned to normal levels in SPF mice by this time point (Fig. 2e, f). This may reflect the increased worm burdens noted at this time point in antibiotic-treated mice (Fig. 1a).

Taken together, these data indicated that mice lacking a complex bacterial microbiota mount a similar, or slightly elevated, type 2 immune response against *Hpb* infection as compared to SPF mice. This aligns with earlier reports of enhanced type 2 immune responses being elicited by allergens^24^ or following infection with the caecal-dwelling helminth *Trichuris muris*^25^ in germ-free compared to SPF mice.

### The microbiota impacts the distribution of infective helminth larvae within the small intestine

**Fig. 3:**
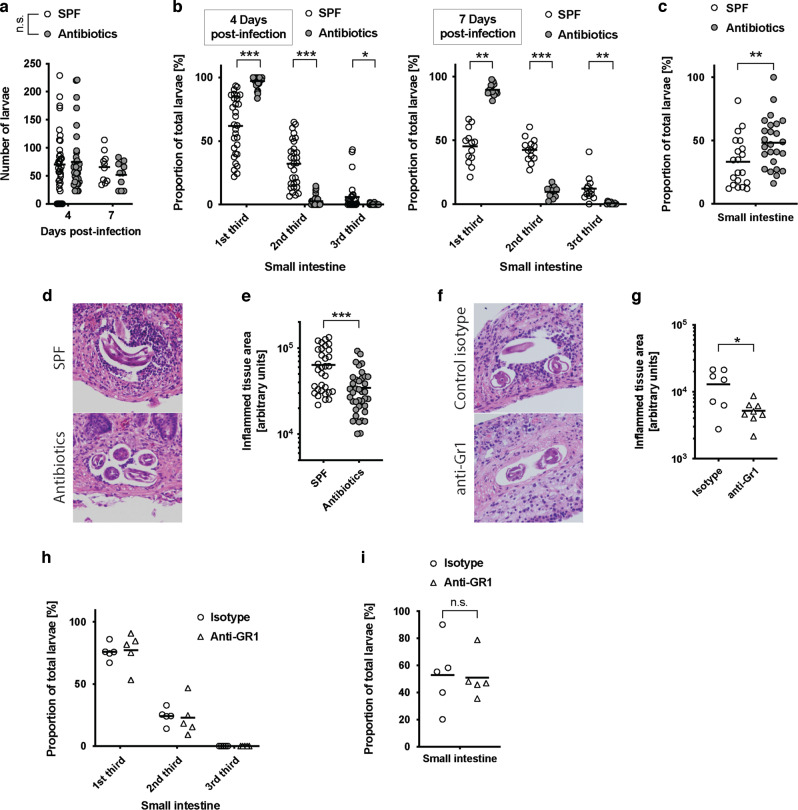
Altered behaviour of *Hpb* larvae in antibiotic-treated mice in not caused by defects in innate immune response in the small intestine. **a**–**i** SPF and antibiotic-treated mice (enrofloxacin for 2 weeks then amoxicillin and clavulanic acid for the rest of the experiment) were infected for 4 or 7 days with 200 *Hpb* larvae. (**a**–**c**) **a** Number of larvae in the intestine was visually assessed and **b** larvae repartition across the length of the intestine reported at the time points shown. **c** Migratory larvae were enumerated at day 4. Data were pooled from at least three independent experiments and analysed using two-way ANOVA. **a**, **b** The number of mice per time point are: (1) For SPF mice D4 = 37 and D7 = 10; (2) for antibiotic-treated mice D4 = 35 and D7 = 10. **c** The number of mice are: (1) for SPF mice D4 = 20; (2) for antibiotic-treated mice D4 = 25. Sections of intestine were (**d**) stained with H&E then (**e**) analysed with ImageJ to quantify the inflamed area around the larvae. At day 4 data from one experiment containing five mice per group were displayed, each dot. The groups were compared using Mann–Whitney. **f**–**i** SPF mice were treated with either anti-Gr1 antibodies or isotype control one day prior and 3 days post-infection. Animals were infected with 200 *Hpb* larvae and a assessed at day 4. Sections of intestine were (**f**) stained with H&E then (**g**) analysed with ImageJ to quantify the inflamed area around the larvae. Data from one experiment containing five mice per group were displayed, each dot represent a larva and its surrounding environment. The groups were compared using Mann–Whitney. **h** Larvae repartition along the intestine was reported and **i** migratory larvae were quantified using Baermann apparatus. Data from one experiment containing five mice per group were displayed and analysed using **h** two-way ANOVA and **i** Mann–Whitney. Each dot represents an individual animal, other than for **e** where each dot represents an individual larva and its surrounding environment. * display statically significant differences between groups.

We next asked whether alterations to *Hpb* larval invasion or development could explain the increased numbers of adult worms present in microbiota-depleted mice. The number of larvae present within the intestinal wall were evaluated at a number of time points and found to be similar between antibiotic-treated and SPF mice (Fig. 3a). By comparison, in the second microbiome-helminth model, we observed that germ-free mice harboured significantly increased numbers of larvae than SPF mice (Fig. S3A). This prompted us to perform more a detailed analysis of how the microbiota could be regulating host resistance against larval invasion. To do this, we compared the intestinal location and migratory ability of larvae present in antibiotic-treated and SPF mice. Infective larvae have a preference for invading the proximal regions of the small intestine^22,26^, and in SPF mice, we found that 50–60% of larvae present at day 4 and day 7 post-infection resided within the first third of the intestine, with the remaining larvae found predominately within the second sections and only a few within the third section (Fig. 3b). In antibiotic-treated mice, the proportion of larvae residing within the most proximal region was increased to 90–97%, with very few larvae found in either the second or third sections (Fig. 3b). These findings aligned with an increased proportion of migratory larvae present in antibiotic-treated mice (Fig. 3c). Therefore, bacterial microbiota regulates the distribution of tissue-dwelling larvae within the small intestine.

### The intestinal microbiota contributes to innate immune responses elicited by helminth larvae

Myeloid cells such as monocytes and neutrophils are the first responders following infection or injury, and recent publications have demonstrated a protective role for neutrophils against the larvae of a related helminth, *Nippostrongylus brasiliensis*, as it migrates through the skin^27^ and lung^28^. Given the microbiota contributes to the mobilisation of myeloid cells from the bone marrow during homoeostasis^29^ and following *Listeria* infection^30^, we next investigated the possibility that it contributes to innate responses raised against *Hpb* larvae. Indeed, antibiotic treatment resulted in a significant reduction in the numbers of *Hpb*-induced monocytes (Fig. S3B), granulocytes (Fig. S3C) and lymphocytes (Fig. S3D) that are present within the circulation. It also reduced the size of inflammatory loci, rich in monocytes and neutrophils, which typically surround the *Hpb* larvae that dwell within intestinal wall (Fig. 3d, e).

We therefore determined the possibility that altered myeloid cell recruitment to the site of larval invasion could explain our observation of increased numbers of migratory larvae following infection of antibiotic-treated mice. For this purpose, *Hpb* infected SPF mice were treated with anti-Gr1 monoclonal antibodies (mAb) to deplete circulating neutrophils and monocytes. As expected, anti-Gr1 mAb treatment of mice, just prior to and during *Hpb* infection, resulted in a reduction of myeloid cells to the inflammatory loci surrounding the larvae, akin to that observed in antibiotic-treated mice (Fig. 3f, g). Surprisingly however, anti-Gr1 mAb treatment did not affect the distribution (Fig. 3h) or migratory capacity (Fig. 3i) of the larvae in intestine of SPF mice. Altogether, these results demonstrate that the early mobilisation of innate immune cells in response to *Hpb* infection is strengthened by the presence of a bacterial microbiota but has little impact on the intestinal distribution or migratory capacity of *Hpb* larvae.

### Microbiota-driven intestinal motility impacts on larval site selection within the small intestine and their migratory capacity in a Baermann assay

**Fig. 4:**
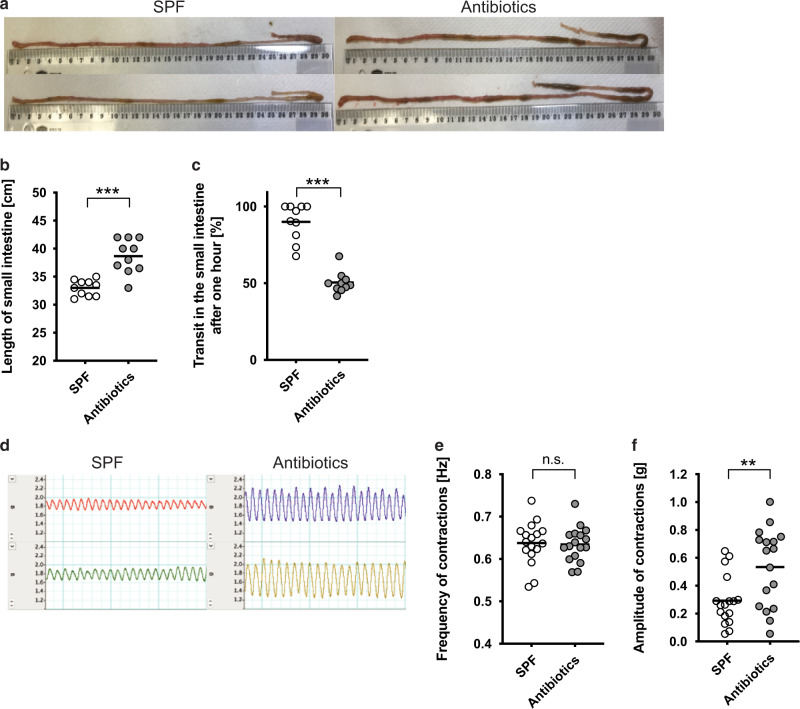
Antibiotic-treated mice showed a reduced peristalsis and an increased amplitude of spontaneous intestinal muscle contractions. **a**–**f** Mice were treated with antibiotics (enrofloxacin for 2 weeks then amoxicillin and clavulanic acid for the rest of the experiment) or saline vehicle? **a**, **b** The length of the small intestine was measured using a ruler. **a** Represent intestines from two different mice of each group. **b** Data from two independent experiment were pooled (*n* = 10) and analysed using Mann–Whitney. **c** Transit was measured using carmine dye, the percentage of small intestinal length travelled 1 h after gavage was reported. Data from two independent experiment were pooled (*n* = 10) and analysed using Mann–Whitney. **d**–**f** Intestinal muscle contractions were analysed ex vivo using an organ bath and force transducers. **d** Recorded contractions pattern from two different mice per group are represented. Average (**e**) frequency and (**f**) amplitude were calculated from the contraction pattern recorded. Data were pooled from two independent experiment (*n* = 17) and were analysed using Mann–Whitney. Each dot represents an individual animal. * display statically significant differences between groups.

**Fig. 5:**
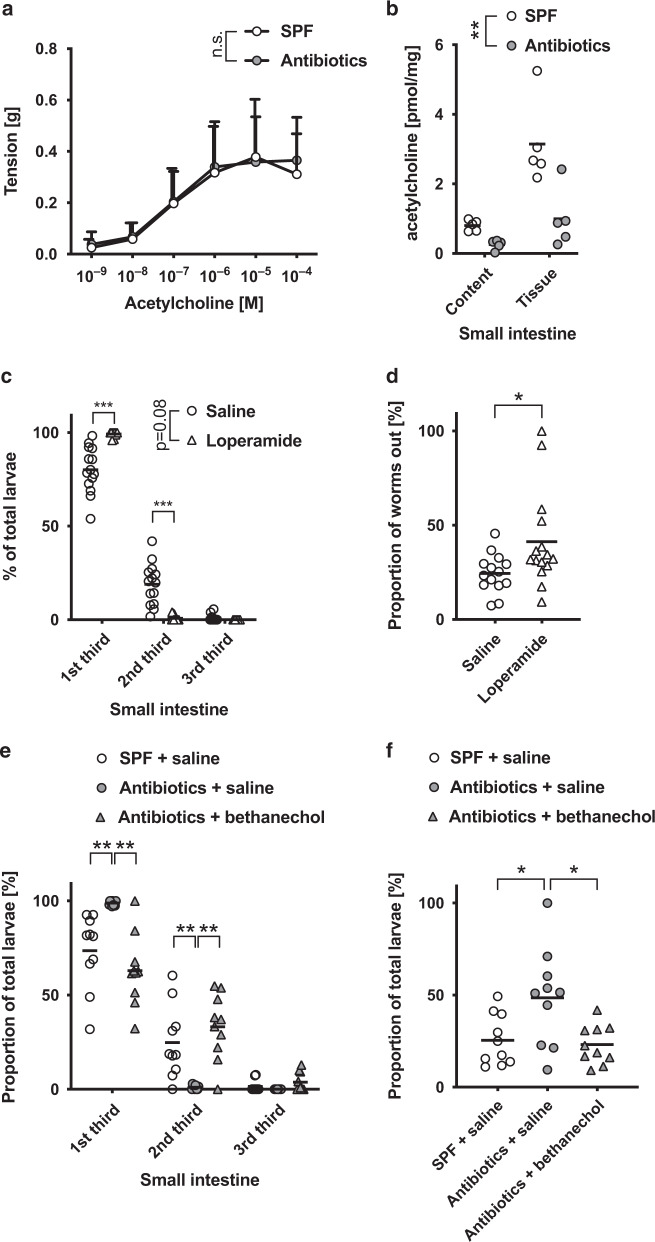
Altered behaviour of *Hpb* larvae in antibiotic-treated mice can be replicated by altering transit speed. **a**, **b** Mice were treated or not with antibiotics (enrofloxacin for 2 weeks then amoxicillin and clavulanic acid for the rest of the experiment). **a** Intestinal muscle contractions were analysed ex vivo using an organ bath and force transducers. Increasing concentration of ACh were used to induce contractions. Data were pooled from two independent experiment (*n* = 17), each dot represents the average of all the mice for each group and were analysed using two-way ANOVA. **b** Choline and ACh were extracted and measured from either intestinal tissue or content of the lumen. Data represents one experiment with five mice per group and were analysed by two-way ANOVA. **c**, **d** SPF mice were treated with either saline or loperamide (200 mg) daily and infected with 200 larvae for 4 days. **c** Repartition across the length of the intestine was reported and **d** migratory larvae were numbered. Data were pooled from three independent experiments (*n* = 14) and analysed using **b** two-way ANOVA or **c** Mann–Whitney. **e**, **f** Mice were treated or not with antibiotics (enrofloxacin for 2 weeks then amoxicillin and clavulanic acid for the rest of the experiment). Antibiotic-treated mice were treated with bethanechol (20 mg) 1 h and 1 day infection. All groups were infected with 200 larvae for 4 days. **e** Repartition across the length of the intestine was reported and **f** migratory larvae were numbered. Data were pooled from two independent experiments (*n* = 10) and analysed using **e** two-way ANOVA or **f** Mann–Whitney. Each dot represents and individual animal. * display statically significant differences between groups.

The presence of a complex microbiota has been shown to contribute to colonic motility^31,32^, leading us to speculate that alterations to small intestinal motility may contribute to the altered distribution of *Hpb* larvae in the small intestine. To test this, we first investigated whether the microbiota also contributed to small intestinal transit time using an in vivo assay of dye migration. Antibiotic-treated mice exhibited an elongation of the small intestine (Fig. 4a, b). Surprisingly this was not observed for germ-free mice (Fig. S4A), despite previous studies having reported small intestinal elongation in germ-free compared to colonised PRM/Alf and C3H/He mouse strains^33^. As expected microbial depletion resulted in a significantly slower transit rate for the small intestine of antibiotic-treated mice (Fig. 4c) and germ-free mice (Fig. S4B) compared to SPF mice, even after normalising for the increased intestinal length. Interestingly, the use of an organ bath to investigate contractions in excised jejunal tissue form SPF and antibiotic-treated mice exhibited a similar frequency of spontaneous contractions (Fig. 4d, e), but that tissue from antibiotic-treated mice had an increase in the amplitude of spontaneous contractions (Fig. 4d, f). Similar observations were made in germ-free animals (Fig. S4C, D). We next investigated the responsiveness of the jejunal segments to acetylcholine (ACh), as this represents one of the major excitatory neurotransmitters contributing to muscle contractility and promoting small intestinal motility^34^. No differences were observed in the contractile response of jejunal segments from antibiotic-treated or SPF mice to increasing doses of ACh (Fig. 5a).

This suggested that the expression of excitatory muscarinic receptors, and the resulting tissue response to ACh, is not altered by microbiota depletion. We therefore investigated whether the availability of ACh was altered by antibiotic treatment. The concentration of ACh was strongly decreased in both the intestinal content and the intestinal tissue of antibiotic-treated compared to SPF mice (Fig. 5b). This is in agreement with prior reports suggesting the microbiota may act as a potential source of ACh^35^, and additional raises the possibility that microbial signals promote host ACh production. It is also likely that the reduced ACh availability, at least in part, underlies the altered transit time in the small intestine noted in those mice lacking a complex microbiota.

To determine whether the observed contribution of the intestinal microbiota to small intestinal transit contributed to altered larval distribution observed following *Hpb* infection of antibiotic-treated mice, we employed the use of drugs commonly used to promote or inhibit intestinal motility. Loperamide, also known as Imodium, is commonly used to treat diarrhoea due to its ability to reduce intestinal contractions. To mimic the ability of antibiotic treatment to slow small intestinal transit, SPF mice were treated with loperamide and simultaneously infected with *Hpb*. Loperamide treatment did not alter the number of *Hpb* present within the intestinal wall but significantly altered the distribution of larvae along the small intestine (Fig. 5c), as compared to saline-treated mice. Larvae in loperamide-treated mice also exhibited increased migration in a Baermann assay compared to saline-treated mice (Fig. 5d). These data resembled those collected from antibiotic-treated mice indicating that reduced intestinal motility in these mice may indeed contribute to the observed alterations in *Hpb* larvae. To fully confirm an association between microbial-induced intestinal motility and altered *Hpb* larval behaviour, we next subjected antibiotic-treated mice to additional treatment with bethanechol, a muscarinic receptor agonist that would be expected to compensate for the reduced ACh availability observed in these mice. Bethanechol administration in antibiotic-treated mice did not alter the number of *Hpb* present within the intestinal wall but did alter larval distribution along the small intestine (Fig. 5e) and their migration in a Baermann assay (Fig. 5f).

Indeed, *Hpb* infection of antibiotic-treated mice, that also received bethanechol, more closely resembled SPF mice than mice treated with antibiotics alone (Fig. 5f). We also tested a possible direct effect of the drug on *Hpb* migration by incubating L3 larvae together with increasing doses of bethanechol in vitro, followed by a Baermann assay. No significant impact of the drug was noted for larval migration after 30 or 60 min (Fig. S4E, F). This suggests that bethanechol is able to alter *Hpb* distribution within the small intestine due to its ability to increase intestinal transit as opposed to direct actions on the parasitic larvae. Taken together, these data indicate that a complex microbiota is required for optimal intestinal motility, which in turn favours a more distal location of tissue-dwelling larvae within the small intestine and greater numbers of migratory larvae.

## Discussion

The mammalian intestine contains a complex ecosystem, with members of diverse bacterial phyla representing the most abundant organisms present^14^, while helminths represent the largest and most complex organisms. In the current study, we set out to investigate whether bacterial communities influenced host resistance against intestinal helminths infection, using a model of *Hpb* infection in mice lacking complex bacterial communities. Based on previous studies demonstrating a role for the intestinal microbiota in limiting type 2 immune responses^24^, we hypothesised that helminth infection may skew the immune response of antibiotic-treated or germ-free mice towards enhanced type 2 immunity and improved resistance. In support of this hypothesis, previous studies have reported that germ-free mice infected with *Hpb* harbour significantly less adult worms and eggs^36,37^. However, these studies did not report whether helminth infection led to anaerobic bacterial contamination in the germ-free mice utilised, hence did not allow clear conclusions to be drawn. We therefore decided to repeat these initial studies, by using our previously reported system in which infective *Hpb* larvae are hatched on a lawn of auxotrophic HA107 *E. coli* which cannot colonise germ-free mice^37^. Our data demonstrated that *Hpb* can establish a chronic infection in germ-free mice, and that mice lacking a bacterial microbiota exhibited reduced rather than enhanced resistance towards *Hpb*. These findings were replicated using *Hpb* infection of mice subjected to antibiotic treatment both prior to, and during infection. In these experiments, microbiota depletion continued to be associated with increased numbers and persistence of adult worms. This is consistent with a previous study by Reynolds et al.^38^, that reported delayed *H. polygyrus* expulsion in BALB/C mice treated with vancomycin 1 week prior to infection^38^. Although here authors reported a specific correlation between parasite burdens and the presence of bacteria from the *Lactobacillus* genus. While our current study indicates that *Hpb* does not require any intestinal bacteria to form a productive infection, the study of Reynolds et al.^38^, raises the intriguing possibility that under normal conditions *Hpb* may interact with select bacterial species to modulate parasite fitness and/or host resistance.

To address the question of whether intestinal bacteria influenced parasite viability via alterations to host immune responsiveness, we investigated the known mediators of type 2 immunity, including the production of anti-helminthic effectors such as mucins and RELM-β. These experiments revealed the presence of normal, or slightly enhanced, host type 2 immune responses in antibiotic-treated mice infected with *Hpb*. These findings are consistent with previous reports of increased Th2 cytokine levels in MyD88-deficient mice infected with *Hpb* or *T. muris*^39,40^, although for these reports the increased type 2 responses were associated with improved control of infection^39,40^.

Further examination of antibiotic-treated mice revealed that the initial establishment of the helminth as infective tissue migratory larvae. We additionally demonstrated a role for the bacterial microbiota in driving the early mobilisation of myeloid cells from the bone marrow, and for helminth-associated production of local IFNγ. These data indicated that the early response to *Hpb* infection, including IFNγ production, may be driven by an increased translocation of bacterial across the epithelial barrier, a finding that correlated with the observation of a transient increase in epithelial permeability following *Hpb* infection. Hpb-induced epithelial damage has recently been reported by Hu et al.^41^, who additionally identified small proline rich protein 2A (SPRR2A) as a distinct anti-microbial peptide that is upregulated in response to Hpb infection to limit the dissemination of intestinal bacteria to the underlying MLN. An IFNγ signature, together with natural killer (NK) cells recruitment from the circulation, has been previously reported to occur at the time when *Hpb* larvae penetrates the intestinal wall^42^. In this study, NK cells were not found to impact on larval fitness following antibiotic treatment, however the IFNγ response did aid in limiting tissue damage^42^. Moreover, IFNγ has been reported to drive a foetal-like reversion of intestinal stem cells located within the crypts close to penetrative larvae, supporting the hypothesis that the IFNγ response may serve to limit larval-induced tissue damage. Based on these reports, further studies investigating whether microbiota dissemination is necessary for the IFNγ-driven reparative processes following *Hpb* infection, or bacteria contained within the intestinal lumen is sufficient for this process, would be of great interest. We also noted that the intestinal microbiota was necessary to promote the early mobilisation of myeloid cells around tissue-dwelling larvae following *Hpb* infection. This was unlikely to account for altered host resistance in antibiotic-treated mice, as depletion of these cells in SPF mice did not impact on larval number or migration in a Baermann assay. However, whether IFNγ plays a role in this process remains undetermined.

We next investigated whether increased *Hpb* burdens in the absence of a complex microbiota may result from a role for intestinal bacteria in regulating intestinal physiology. Larval forms of *Hpb* preferentially invade the proximal regions of the small intestine^22,26^, with adult worms residing in a similar location following their exit from the tissues^26,43^. Although the exact reasons for this site selection are unknown, hypotheses include the duodenum being a rich source of nutrients, and a potential action of bile as a host cue stimulating larval invasion^44^. We therefore asked whether the presence or absence of a microbiota impacted on the distribution of invasive *Hpb* larvae throughout the small intestine. We found a greater distribution of larvae within the upper two-thirds of the intestinal wall of antibiotic-treated compared to SPF mice, indicating a smaller proportion of larvae are forced to enter the tissue at more distal portions of the small intestine when a complex microbiota is absent. Based on these findings, we explored the possibility that the microbiota affected larval distribution in the small intestine due to changes in motility. Intestinal motility is driven by smooth muscle contractions that are highly co-ordinated by signals from the enteric nervous system (ENS) and is impacted by both the availability of neurotransmitters as well as the presence of inflammatory cytokines^45,46^. The bacterial microbiota has been identified as an important modulator of the ENS; with germ-free mice exhibiting both reduced nerve densities and altered neural excitability within the intestine^47,48^. Based on reports of disrupted neuro-muscular alterations and reduced colonic transit time in microbiota-depleted mice^12,32^, we reasoned similar alterations were likely to be present in the small intestine. Indeed, both antibiotic-treated and germ-free mice exhibited a slower transit time through the small intestine, alongside alterations to spontaneous jejunal smooth muscle contractions. Correlating with this, we noted reduced levels of ACh within the small intestinal lumen and tissue of antibiotic-treated mice. This observation aligns with prior reports of reduced ACh availability in the colonic tissue of germ-free mice^49^.

Bacterial populations are present throughout the small and large intestine but increase in abundance and complexity along a distal axis. Of note *Hpb* infection impacts on bacterial populations at all sites along the intestine contain microbial populations^50^. Exactly how the microbiota impacts on small intestinal ACh levels is unclear but is likely due to local effects. Interestingly, *Lactobacillus* spp, which are most abundant in the small intestine, have been reported to produce Ach^51^ and can also promote the production of ACh by intestinal neurons^52^. However, whether the reduced ACh observed following microbiota depletion results from decreased *Lactobacillus* spp or from an indirect effect of the microbiota on host ACh production is not clear. ACh is the major excitatory neurotransmitter present in the intestine, and plays a major role in helminth-induced increases in smooth muscle contraction^53^ and in the expulsion of the related parasite *Nippostrongylus brasiliensis*^54–56^. The intestinal microbiota also impacts on the levels of other factors able to impact intestinal motility, including serotonin^57^, substance P^58^ and short-chain fatty acids^59,60^. Bile acids are also impacted strongly by the microbiota^61^ and can either increase or decrease motility^62^ and have been proposed to act as host cue stimulating larval invasion^44^. Interestingly, SPF mice exhibit longer and more narrow villi in the upper small intestine^12^, and adult *Hpb* exhibit a preference for attaching to longer villi^43^. Therefore, it is likely that some, or all of these factors, additionally contribute to our observation of altered *Hpb* distribution within in the small intestine of antibiotic-treated mice.

To test for a possible causal link between intestinal motility and the altered distribution of *Hpb* larvae, we stimulated or inhibited small intestinal motility using commonly available drugs. Our data confirmed a contribution of microbiota-induced intestinal motility to both the distribution of *Hpb* larvae in the small intestine and their migration (as assessed by Baermann assay). To our knowledge, this is the first report that provides evidence for a causal link between intestinal motility and worm behaviour for *Hpb*, adding weight to the widely established hypothesis that altered intestinal transit contributes to host resistance against intestinal helminths. Further support to a link between intestinal motility and helminth resistance can also be found in a recent report by Chen et al., who demonstrated a role for IL-33 in promoting intestinal motility and the expulsion of *T. muris* through its ability to stimulate the release of an excitatory neurotransmitter, serotonin, from intestinal enterochromaffin cells^63^.

Based on these studies, we propose an extended model of colonisation resistance to include the ability of the bacterial microbiota to promote resistance against intestinal helminths. Our findings highlight bacterial-induced intestinal motility as a key factor contributing to host resistance. However, it is likely that this forms only one of several mechanisms by which the bacterial microbiota may influence intestinal physiology to alter the course of intestinal helminth infection. It is also likely that direct interactions between bacteria and helminths contribute to both the establishment and chronicity of these pathogens in the mammalian intestine as recently demonstrated for *T. muris*^14^. In summary, our findings reveal that the bacterial intestinal microbiota alters intestinal motility and negatively impacts on the ability of infective helminth larvae to invade intestinal tissue at their preferred site, with a subsequent reduction in the burden and chronicity of the adult worms.

## Materials and methods

### Mouse housing and license

C57BL/6 wild-type mice were purchased from Charles River Laboratories or Monash Animal Research Platform. All mice were maintained under SPF conditions at the École Polytechnique Fédérale de Lausanne (EPFL), Switzerland or by the Monash Intensive Care Unit Facility at Monash University, Alfred Research Alliance, Melbourne, Australia. All animal experiments were approved by the Service de la consommation et des affaires vétérinaires (1066 Épalinges, Canton of Vaud, Switzerland), or the Alfred Research Alliance Animal Ethics Committee, Melbourne, Australia. Germ-free mice were purchased from the University of Bern and housed in flexible-film isolators at the EPFL.

### Antibiotic-treated mice and sterility tests

We used the antibiotic treatment regime that was developed by our laboratory^16,21^. Briefly, mice were treated with 2.5 mg/ml enrofloxacin in drinking water for 2 weeks, followed by 0.8 mg/ml of amoxicillin and 0.114 mg/ml clavulanic acid in drinking water for a minimum of 2 additional weeks prior to infection. Treatment with amoxicillin and clavulanic acid was continued throughout the experiment. During the period of antibiotic treatment, drinking water was renewed 3 times a week (every 2–3 days). The sterility of germ-free mice and the bacterial depletion of antibiotic-treated mice were assessed by plating caecal content on culture plates kept under aerobic and anaerobic conditions as well as by caecal smears analysis, as previously described^16,21^.

### Intestinal transit assay and medication

The protocol to assess intestinal transit time was adapted from publication^64^. Briefly, 250 µl of a solution made with 60 mg/ml carmine (Sigma-Aldrich C1022) and 5 mg/ml methylcellulose (Sigma-Aldrich 274429) in saline was orally gavaged in mice. After 1 h small intestines were collected and both the total length and the progression of the red colourant were measured using a ruler. Transit is displayed as the percentage of the small intestine length the dye had travelled. The pharmacological treatment used to reduce intestinal transit was 2 mg/ml of loperamide hydrochloride (Sigma-Aldrich PHR1162) in 100 µl saline, orally gavage daily from 0 to 4 days post-infection. To restore transit speed in antibiotic-treated mice, 10 mg/ml bethanechol (Sigma-Aldrich C5259) in 200 µl saline was administrated by oral gavage 1 h before infection and 1 day after infection. For in vitro assays 200 *Hpb* L3 larvae were incubated with the indicated doses of bethanechol in saline at 37 °C for 10 min and then migration was assessed with a modified Baermann apparatus.

### Helminth cycle and infection

The standard *Hpb* infective stage-three larvae (L3) were generated in the laboratory based on published protocols^19^. Antibiotic-washed *Hpb* larvae were generated using successive washings with PBS and RPMI containing enrofloxacin (5 mg/ml), amoxicillin (2 mg/ml), and clavulanic acid (0.2 mg/ml)^16,21^. To generate axenic larvae, *Hpb* adults were recovered from mice infected for 21 days using a modified Baermann apparatus. After 3 h of incubation adult worms were collected and washed three times with sterile saline containing 0.2 mg/ml gentamycin (Sigma-Aldrich G1397), 200 units/ml penicillin and 0.2 mg/ml streptomycin (Sigma-Aldrich P4333). Worms were then washed with RPMI (Gibco 21875-034) containing the same antibiotics and cultured overnight at 37 °C in RPMI containing 0.1 mg/ml gentamycin, 100 units/ml penicillin, 0.1 mg/ml streptomycin and 10% glucose. The next day, eggs were collected, centrifuged at 800 relative centrifugal force (RCF) for 7 min, resuspend in autoclaved water and incubated on sterile ‘nematode growth media’ agar plates in the presence of an auxotrophic *Escherichia coli* strain HA107^65^. The plates were placed in plastic boxes containing wet paper for humidity and incubated at 27 °C for 4–5 days. The L3 infective larvae were then collected in sterile saline and stored at 4 °C until needed for infection of germ-free mice (dose of 400 larvae/mouse). The sterility of eggs and larvae was tested by culture on aerobic and anaerobic agar plates (as described above for germ-free mice) and batches without contamination were used.

### Worm number and migratory ability

Once the animals were humanely euthanised, the number and distribution of worms in the tissue were counted. The small intestine was harvested, immersed in saline solution at room temperature and then opened longitudinally with scissors, before being mounted between two large glass slides (20 cm × 20 cm). Larvae or adult worms were then counted under a stereomicroscope. Section of intestines were determined by dividing the small intestines into three equal parts and referred to in the text as thirds. The ability of larvae to migrate out of the tissue at 4 days post-infection, or following drug treatment in vitro, was assessed with a modified Baermann apparatus as follows: after counting the larvae in the tissue, the small intestine was hung over on a slim strip of laboratory film (Parafilm) placed over a 50-ml falcon tube containing saline. The falcon tube was then incubated in a 37 °C water bath for 3 h. At the end of the incubation, the 6 ml at the bottom of the tube were transferred in a 10-cm Petri dish and worms were counted under a stereomicroscope^19^. The production of eggs was measured in the faeces (time-course) or in the caecal content. The faecal or caecal material was weighted in a 15-ml tube and dissolved in 3 ml saline; before counting, 3 ml of saturated NaCl solution was added and agitated by vortex. From the top, 500 µl of volume was transferred to a McMaster counting slide. The eggs were finally counted in a volume of 150 µl under a stereomicroscope.

### Intracellular cytokine staining and flow cytometry

The total helper T-cell responses were assessed by flow cytometry. MLNs were crushed, and the absolute number of cells was counted with a Guava Personal Cell Analysis flow cytometer (Merck Millipore) using the Guava viability reagent (Merck Millipore 4000-0041). Stimulation of 10^5^ cells was performed with 10^–7^ mM phorbol myristate acetate (PMA, Invivogen tlrl-pma), 1 µg/ml ionomycin (Biotrend BN0275) and 2 µM monensin (Sigma-Aldrich M5273) in complete RPMI containing 10% foetal bovine serum (Gibco 10270-106), 10 mM HEPES, 100 units/ml penicillin, 0.1 mg/ml streptomycin and 0.05 beta-mercaptoethanol, for 3 h at 37 °C. Cells were stained with the Aqua fluorescent dead cells stain (Invitrogen, L34957) in flow cytometry buffer (saline with 0.2–0.5 % bovine serum albumin); then with anti-CD4-PB (Biolegend 100428). Intracellular stainings were performed with Foxp3 staining buffer set (Ebioscience 00-5523-00) and with anti-IFNγ-FITC (Biolegend 505806), anti-IL4-PE (Biolegend 504104) and anti-IL13-biotin (Ebioscience 13-7135-85) overnight. Streptavidin-PECy7 (Biolegend 405206) was used to stain anti-IL-13-biotin antibodies. The samples were measured on an LSRII flow cytometer (Becton Dickinson) and analysed with Flowjo software version 9.

### Antibody quantification (ELISA)

Mouse tail bleeds were performed and the collected serum was centrifuged according to the instructions of the manufacturer (2–3 min at 6000–15,000 RCF). Serum samples were then stored at −80 °C and quantified by enzyme-linked immuno-sorbent assay (ELISA) as previously reported^7^. Briefly, on day one, Nunc Maxisorp 96-well plates were coated with either 1 µg/ml anti-IgE (Biolegend 406902), 5 µg/ml anti-IgG1 (Southern Biotech 1070-01), 5 µg/ml anti-IgG2a/c (Southern Biotech 1080-01) or 1–10 µg/ml of excretory-secretory products from adult *H. polygyrus* in ELISA coating buffer (Ebioscience 00-0044-59), and incubated overnight at 4 °C. On day 2, plates were blocked with 2.5% bovine serum albumin (Roche 10 735 108 001) in saline. Then, serially dilutedserum samples as well as IgE (Biolegend 401801), IgG1 (Southern Biotech 0102-01) or IgG2a/c (Southern Biotech 0103-01) standards were added (diluted in saline with 0.1% bovine serum albumin) and incubated overnight at 4 °C. The concentration of *Hpb*-specific antibodies was established by comparing with an internal standard made from pooled serum from mice after secondary infection. On day 3, 1 µg/ml alkaline-phosphatase-conjugated anti-IgE (Southern Biotech 1130-04), anti-IgG1 (Southern Biotech 1070-06) or anti-IgG2a/c (Southern Biotech 1080-04) was added and incubated for 1 h. Finally, 1 mg/ml of 4-nitrophenyl phosphate sodium salt hexahydrate (Sigma-Aldrich 71770) was used as a substrate in diethanolamine buffer and the colorimetric reaction was read at 405 nm on a Benchmark Plus spectrophotometer (Bio-Rad Laboratories).

### Cytokine quantification

Peritoneal lavage was performed with 1 ml of saline injected in the peritoneal cavity. For each mouse, 800 µl of liquid was recovered from the peritoneal cavity. IL-4, IL-5, IL-13 and IFNγ were then quantified in serum and peritoneal wash samples multiplex kits (Ebioscience) on a Luminex 100/200 instrument (Luminex Corporation) according to the manufacturer instructions.

### Histology and immunofluorescence

Small “Swiss-rolled” intestinal sections (4 µm) were cut mounted and either stained with Alcian blue and periodic acid-Schiff (PAS, mucus) or left unstained for further processing. Staining of RELM-β was adapted from a published method^66^. Sections were stained with primary antibodies, anti-RELM-β antibody (Peprotech 500-P215) or control IgG, followed by incubation with secondary antibody, anti-rabbit IgG biotinylated (Jackson Immunoresearch 711-065-152). Vectastain peroxidase kit (Vector Laboratories PK-6100) and 3,3’-diaminobenzidine were used to reveal the staining and counter-stained with Harris’ solution. The total number of goblet cells in the intestinal epithelial lining was determined by counting the cells immunostained with PAS, and based on their morphology and location being consistent with goblet cells, in one random high-power field (magnification) displaying mucosal integrity and no artefacts. Tuft cells staining was performed using anti-DCLK1 antibody (Abcam, AB31704) and anti-rabbit Alexa Fluor 488 (Jackson ImmunoResearch, 111-545-003). Nuclei were then fluorescently labelled using NucBlue (Invitrogen, Hoechst 33342). The number of tuft cells per villi were counted in five random high-power fields (what magnification) per sample displaying mucosal integrity and no artefacts. These values should be taken as relative differences rather than absolute levels.

### Neutrophils and monocytes depletion

Anti-GR1 (Bio X Cell, clone RB6-8C5) or a rat IgG2b isotype control (Bio X Cell, clone LTF2) antibodies were injected intraperitoneally from 1 to 3 days post-infection (0.25 mg per mouse and per day), in saline. The efficacy of the depletion was assessed by histology and flow cytometry (data not shown).

### Recruitment quantification

The cell density and inflamed tissue area around the tissue-dwelling larvae were assessed using intestinal “Swiss rolls” sections stained with haematoxylin and eosin. Photographs were taken on a bright-field microscope and analysed with the image-processing software FIJI 2.3.0. The region of interest (inflamed area) around intestinal granulomas was manually drawn and the nuclei staining area within it, were quantified by FIJI using colour deconvolution and Huang threshold.

### White blood cells counts

About 50 μl of blood were collected and processed on a haematology analyser (SCIL Vet Abc), according to manufacturer’s instructions (SCIL Animal Care).

### Intestinal contractility ex vivo

The intestinal transit in vivo was described above. The organ bath analysis ex vivo was based on published protocols^53^. Briefly, two longitudinal samples of about 2 cm each were dissected from the first third of the small intestine (centimetres 6–8 and 8–10, section above the first 5 cm were considered as jejunum) and immersed in the vessels of the organ bath (Panlab Harvard Apparatus) filled with Krebs buffer at 37 °C and bubbling of carbogen (95% O_2_ and 5% CO_2_). One end of the intestinal samples was attached to a fixed holder and the other to a force transducer (AD Instruments). A baseline tension of 1 g was set followed by an equilibration period of 1 h with buffer change every 15 min throughout the experiment. Then, increasing concentration of ACh (10^−10^ to 10^−3^ M) were applied to the tissues every minute. The maximum tension after each concentration of ACh was used to build a dose response curve to analyse ACh-induced contractions of the tissues. The tension (spontaneous and induced contractions) of the tissue was recorded and analysed with the software Labchart (AD Instruments).

### Acetylcholine measurement

Samples of the first, second and third section of the small intestine from either from the tissue or the lumen were processed using Choline/Acetylcholine Assay Kit (Abcam, ab65345). Lumen samples were collected by gently scraping the small intestine with a coverslip to recover content and mucus on the surface of the tissue. The resulting “scraped” small intestinal tissues were used in subsequent analysis. All samples used for ACh quantification was kept on ice to prevent ACh degradation. Samples were processed as per manufacturer’s instructions. If necessary, tissue was lysed using beads and then incubated in buffer to extract ACh and choline. All samples were incubated with or without choline esterase in the mix to measure the amount of ACh. The kit measures the amount of choline present in the sample using enzymatic reaction with colorimetric reading Therefore, the amount of ACh in each sample was deduced from the measurements with and without choline esterase.

### Intestinal permeability

The intestinal permeability was assessed with fluorescein isothiocyanate-dextran (FITC-dextran) based on established protocols^67^. Water and food were removed from the cage and 10 mg of FITC-dextran, with a molecular weight of 4000 daltons (Sigma-Aldrich 46944), was orally gavaged in a volume of 200 μl of saline (Gibco 10010-015) per mouse. Four hours after gavage, mice were humanely euthanised and cardiac bleeds were performed. The blood was collected in serum tubes (BD Biosciences 365968) and centrifuged 3 min at 10,000 RCF. It was then prepared in a flat-bottom 96-well plate, together with a standard diluted in saline, and analysed on a plate reader (Safire 2, Tecan) with excitation at 485/20 nm and emission at 528/20 nm.

### Data presentation and statistics

The bars are the mean and the error bars are the standard deviation. Two groups are compared with Mann–Whitney *U* tests. Repeated measurements of a single group are tested with Kruskal–Wallis tests. Multiple groups are compared with one-way analysis of variance (ANOVA) followed by Tukey’s multiple comparison test or two-way ANOVA followed by Sidak’s multiple comparisons test.

*p* values higher than 0.05 were considered as non-significant and abbreviated “n.s.”. *p* values lower than or equal to 0.05, 0.01 and 0.001 are respectively represented by one, two or three asterisks (*) or hashes (#). We used asterisks (above a horizontal bar) to show the comparison between groups at a given time-point and hashes (below a horizontal bar) to show the comparison, within a group, to the earliest time-point (usually naïve mice). The statistical analyses were performed with GraphPad Prism 8 (GraphPad Software).

## Supplementary Information

Supplementary Material

## References

[1] Behnke J, Barnard C, Wakelin D (1992). Understanding chronic nematode infections: evolutionary considerations, current hypotheses and the way forward. Int. J. Parasitol..

[2] Araujo A, Reinhard KJ, Ferreira LF, Gardner SL (2008). Parasites as probes for prehistoric human migrations?. Trends Parasitol..

[3] Fumagalli M (2009). Parasites represent a major selective force for interleukin genes and shape the genetic predisposition to autoimmune conditions. J. Exp. Med..

[4] Cardoso V (2017). Neuronal regulation of type 2 innate lymphoid cells via neuromedin U. Nature.

[5] Anthony R, Rutitzky L, Urban J, Stadecker M, Gause W (2007). Protective immune mechanisms in helminth infection. Nat. Rev. Immunol..

[6] Harris N, Pleass R, Behnke J (2014). Understanding the role of antibodies in murine infections with *Heligmosomoides* (*polygyrus*) *bakeri*: 35 years ago, now and 35 years ahead. Parasite Immunol..

[7] McCoy K (2008). Polyclonal and specific antibodies mediate protective immunity against enteric helminth infection. Cell Host Microbe.

[8] Shea-Donohue T (2001). The role of IL-4 in *Heligmosomoides polygyrus*-induced alterations in murine intestinal epithelial cell function. J. Immunol..

[9] Hasnain SZ, Thornton DJ, Grencis RK (2011). Changes in the mucosal barrier during acute and chronic Trichuris muris infection. Parasite Immunol..

[10] Herbert DR (2009). Intestinal epithelial cell secretion of RELM-beta protects against gastrointestinal worm infection. J. Exp. Med..

[11] Sommer F, Bäckhed F (2013). The gut microbiota—masters of host development and physiology. Nat. Rev. Microbiol..

[12] Smith K, McCoy K, Macpherson A (2007). Use of axenic animals in studying the adaptation of mammals to their commensal intestinal microbiota. Semin. Immunol..

[13] Spees A, Lopez C, Kingsbury D, Winter S, Bäumler A (2013). Colonization resistance: battle of the bugs or ménage à trois with the host?. PLoS Pathog..

[14] Rapin A, Harris NL (2018). Helminth-bacterial interactions: cause and consequence. Trends Immunol..

[15] McInnes IB (2003). A novel therapeutic approach targeting articular inflammation using the filarial nematode-derived phosphorylcholine-containing glycoprotein ES-62. J. Immunol..

[16] Zaiss M (2015). The intestinal microbiota contributes to the ability of helminths to modulate allergic inflammation. Immunity.

[17] Brosschot TP (2021). Impaired host resistance to Salmonella during helminth co-infection is restored by anthelmintic treatment prior to bacterial challenge. PLoS Neglected Tropical Dis..

[18] Harris N (2011). Advances in helminth immunology: optimism for future vaccine design?. Trends Parasitol..

[19] Camberis, M., Le Gros, G. & Urban, J. Jr. Animal Model of Nippostrongylus brasiliensis and Heligmosomoides polygyrus. *Curr. Protoc. Immunol.*, **55**, 19.12.1–19.12.27. (2003). 10.1002/0471142735.im1912s55. 10.1002/0471142735.im1912s55 18432905

[20] Reynolds L, Filbey K, Maizels R (2012). Immunity to the model intestinal helminth parasite *Heligmosomoides polygyrus*. Semin. Immunopathol..

[21] Mosconi I (2015). Parasite proximity drives the expansion of regulatory T cells in Peyer’s patches following intestinal helminth infection. Infect. Immun..

[22] Panter H (1969). Host-parasite relationships of *Nematospiroides dubius* in the mouse. J. Parasitol..

[23] Esser-von Bieren J (2015). Antibody-mediated trapping of helminth larvae requires CD11b and Fcgamma receptor I. J. Immunol..

[24] Herbst T (2011). Dysregulation of allergic airway inflammation in the absence of microbial colonization. Am. J. Respiratory Crit. Care Med..

[25] Hayes KS (2010). Exploitation of the intestinal microflora by the parasitic nematode Trichuris muris. Science.

[26] Lewis J, Bryant V (1976). The distribution of *Nematospiroides dubius* within the small intestine of laboratory mice. J. Helminthol..

[27] Bouchery T (2020). Hookworms evade host immunity by secreting a deoxyribonuclease to degrade neutrophil extracellular traps. Cell Host Microbe.

[28] Ajendra J (2020). IL-17A both initiates, via IFNgamma suppression, and limits the pulmonary type-2 immune response to nematode infection. Mucosal Immunol..

[29] Balmer ML (2014). Microbiota-derived compounds drive steady-state granulopoiesis via MyD88/TICAM signaling. J. Immunol..

[30] Khosravi A (2014). Gut microbiota promote hematopoiesis to control bacterial infection. Cell Host Microbe.

[31] Anitha M, Vijay-Kumar M, Sitaraman S, Gewirtz A, Srinivasan S (2012). Gut microbial products regulate murine gastrointestinal motility via toll-like receptor 4 signaling. Gastroenterology.

[32] Muller P (2014). Crosstalk between muscularis macrophages and enteric neurons regulates gastrointestinal motility. Cell.

[33] Slezak K (2014). Association of germ-free mice with a simplified human intestinal microbiota results in a shortened intestine. Gut Microbes.

[34] Costa M, Brookes SJ, Hennig GW (2000). Anatomy and physiology of the enteric nervous system. Gut.

[35] Cryan JF, Dinan TG (2012). Mind-altering microorganisms: the impact of the gut microbiota on brain and behaviour. Nat. Rev. Neurosci..

[36] Chang J, Wescott R (1972). Infectivity, fecundity, and survival of *Nematospiroides dubius* in gnotobiotic mice. Exp. Parasitol..

[37] Rausch S (2018). Parasitic nematodes exert antimicrobial activity and benefit from microbiota-driven support for host immune regulation. Front. Immunol..

[38] Reynolds L (2014). Commensal-pathogen interactions in the intestinal tract: Lactobacilli promote infection with, and are promoted by, helminth parasites. Gut Microbes.

[39] Reynolds, L. V. et al. MyD88 signaling inhibits protective immunity to the gastrointestinal helminth parasite Heligmosomoides polygyrus. *J. Immunol.* **193**, 2984–2993 (2014). 10.4049/jimmunol.1401056 PMC4157852 25114104

[40] Helmby H, Grencis R (2003). Essential role for TLR4 and MyD88 in the development of chronic intestinal nematode infection. Eur. J. Immunol..

[41] Hu Z (2021). Small proline-rich protein 2A is a gut bactericidal protein deployed during helminth infection. Science.

[42] Gentile ME (2020). NK cell recruitment limits tissue damage during an enteric helminth infection. Mucosal Immunol..

[43] Bansemir A, Sukhdeo M (1996). Villus length influences habitat selection by *Heligmosomoides polygyrus*. Parasitology.

[44] Sukhdeo M, Croll N (1981). The location of parasites within their hosts: bile and the site selection behaviour of *Nematospiroides dubius*. Int. J. Parasitol..

[45] Akiho H, Blennerhassett P, Deng Y, Collins S (2002). Role of IL-4, IL-13, and STAT6 in inflammation-induced hypercontractility of murine smooth muscle cells. Am. J. Physiol. Gastrointest. Liver Physiol..

[46] Akiho H, Ihara E, Motomura Y, Nakamura K (2011). Cytokine-induced alterations of gastrointestinal motility in gastrointestinal disorders. World J. Gastrointest. Pathophysiol..

[47] Collins J, Borojevic R, Verdu E, Huizinga J, Ratcliffe E (2014). Intestinal microbiota influence the early postnatal development of the enteric nervous system. Neurogastroenterol. Motil..

[48] McVey Neufeld K, Mao Y, Bienenstock J, Foster J, Kunze W (2013). The microbiome is essential for normal gut intrinsic primary afferent neuron excitability in the mouse. Neurogastroenterol. Motil..

[49] De Palma G (2015). Microbiota and host determinants of behavioural phenotype in maternally separated mice. Nat. Commun..

[50] Rapin A (2020). Infection with a small intestinal helminth, Heligmosomoides polygyrus bakeri, consistently alters microbial communities throughout the murine small and large intestine. Int. J. Parasitol..

[51] Roshchina, V. V. New Trends and Perspectives in the Evolution of Neurotransmitters in Microbial, Plant, and Animal Cells. *Adv. Exp. Med. Biol*. **874**, 25–77 (2016). 10.1007/978-3-319-20215-0_2 26589213

[52] Chandrasekharan B (2019). Interactions between commensal bacteria and enteric neurons, via FPR1 induction of ROS, increase gastrointestinal motility in mice. Gastroenterology.

[53] Zhao A (2003). Dependence of IL-4, IL-13, and nematode-induced alterations in murine small intestinal smooth muscle contractility on Stat6 and enteric nerves. J. Immunol..

[54] Darby M (2015). The M3 muscarinic receptor is required for optimal adaptive immunity to helminth and bacterial infection. PLoS Pathog..

[55] Roberts, L. B. et al. Acetylcholine production by group 2 innate lymphoid cells promotes mucosal immunity to helminths. *Sci. Immunol.* **6** eabd0359 (2021). 10.1126/sciimmunol.abd0359 33674321

[56] Chu, C. et al. The ChAT-acetylcholine pathway promotes group 2 innate lymphoid cell responses and anti-helminth immunity. *Sci. Immunol.* **6** eabe3218 (2021). 10.1126/sciimmunol.abe3218 PMC8577047 33674322

[57] Yano JM (2015). Indigenous bacteria from the gut microbiota regulate host serotonin biosynthesis. Cell.

[58] Liljedahl S, Mattsson O, Pernow B (1958). The effect of substance P on intestinal motility in man. Scand. J. Clin. Lab. Investig..

[59] Dass N (2007). The relationship between the effects of short‐chain fatty acids on intestinal motility in vitro and GPR43 receptor activation. Neurogastroenterol. Motil..

[60] Tazoe H (2008). Roles of short-chain fatty acids receptors, GPR41 and GPR43 on colonic functions. J. Physiol. Pharm..

[61] Sayin SI (2013). Gut microbiota regulates bile acid metabolism by reducing the levels of tauro-beta-muricholic acid, a naturally occurring FXR antagonist. Cell Metab..

[62] Zhan, K. et al. Gut microbiota-bile acid crosstalk in diarrhea-irritable bowel syndrome. *BioMed Res. Int.* **2020**, 3828249 (2020). 10.1155/2020/3828249 PMC7676935 33274207

[63] Chen Z (2021). Interleukin-33 promotes serotonin release from enterochromaffin cells for intestinal homeostasis. Immunity.

[64] Li Z (2011). Essential roles of enteric neuronal serotonin in gastrointestinal motility and the development/survival of enteric dopaminergic neurons. J. Neurosci..

[65] Hapfelmeier S (2010). Reversible microbial colonization of germ-free mice reveals the dynamics of IgA immune responses. Science.

[66] Herbert D (2009). Intestinal epithelial cell secretion of RELMβ protects against gastrointestinal worm infection. J. Exp. Med..

[67] Ibla JC, Khoury J (2013). Methods to assess tissue permeability. Methods Mol. Biol..

