# Supplementary material for: Microbial regulation of intestinal motility provides resistance against helminth infection
Source: Mucosal Immunol. 2022 Mar 14;15(6):1283–95. doi: 10.1038/s41385-022-00498-8 (PMC9705251; doi:10.1038/s41385-022-00498-8)
Supplement: Supplementary file 1 — Supplementary Material [file 41385_2022_498_MOESM1_ESM.pdf]

## Supplementary Material:

# Microbial regulation of intestinal motility provides resistance against helminth infection

Mati Moyat[1,3,¶], Luc Lebon[1,¶], Olaf Perdijk[3], Lakshanie C. Wickramasinghe[3], Mario M. Zaiss[1,2], Ilaria Mosconi[1], Beatrice Volpe[1], Nadine Guenat[1], Kathleen Shah[1], Gillian Coakley[3], Tiffany Bouchery[1,3], Nicola Harris[1,3,*]

[1] Global Health Institute, Swiss Federal Institute of Technology (EPFL), Lausanne, 1015 Lausanne, Switzerland

[2] Department of Internal Medicine 3, Rheumatology and Immunology, Friedrich-Alexander-University Erlangen-Nürnberg (FAU) and Universitätsklinikum Erlangen, Erlangen, Germany

[3] Department of Immunology and Pathology, Central Clinical School, Monash University, The Alfred Centre, Melbourne, Victoria, Australia

* Corresponding author: Nicola.harris@monash.edu

¶ Equal contribution

## Supplementary Methods:

### Bacterial DNA isolation from ileum and caecum

Caecum and ileum samples (last third of small intestine) were collected into sterile 1.5-ml Biopure tubes (Eppendorf) and directly put on dry ice and stored at −80 °C. Bacterial DNA was isolated using the QiaAMP PowerFecal DNA kit (QIAGEN) according to the manufacturer's instructions.

### 16S rRNA gene library preparation and sequencing.

The V1 and V2 hypervariable regions of the 16S rRNA gene were amplified as described previously [1]. In short, PCRs were performed using an AccuPrime Taq DNA polymerase high fidelity kit (Invitrogen) using the following primers: 5′-**AATGATACG GCGACCACCGAGATCTACAC***TATGGTAATT*CCAGMGTTYGATYMTGGCTCAG-3′ and 5′-**CAAGCAGAAGACGGCATACGAGAT**NNNNNNNNNNNN*AGTCAGTCAGAAG*CTGCCT CCCGTAGGAGT-3′ (bold: Illumina adaptor sequences, italic: linkers, NNNNNNNNNNNN, sample-specific MID tag barcodes). The PCR was run as follows: 3 min at 94 °C for initial denaturation, 30 cycles for cecal samples or 40 cycles for ileal samples of 30 s at 94 °C, 30 s at 56 °C, 1 min 30 s at 72 °C and a 5 min at 72 °C for the final extension step. The quantity and quality of the amplicon products was assessed using the Fragment Analyzer 5200. The products were pooled in equimolar amounts and purified using Agencourt AMPure XP magnetic beads (Beckman Coulter). Sequencing was performed on an Illumina MiSeq platform with MiSeq reagent kit V2-500 (paired-end, 2 × 250).

### 16S rRNA sequencing analysis.

Data analysis was performed in R statistical software. Raw fastq files were demultiplexed and processed using a custom microbiome-dada2 pipeline (https://github.com/respiratory-immunology-lab/microbiome-dada2) with default parameters. Taxonomic classification and exact sequence matching were performed using the SILVA database (version 132). ASV filtering, normalization, ordination and diversity analyses were performed using the phyloseq R package (version 1.26.1) and visualized using the ggplot2 R package (version 3.1.0). Only samples with >500 ASVs were considered for downstream analyses. Unclassified ASVs at

the phylum level were removed and filtered based on counts (5 reads minimum) and the count table was then normalized using total sum scaling.

## Supplementary Figures:

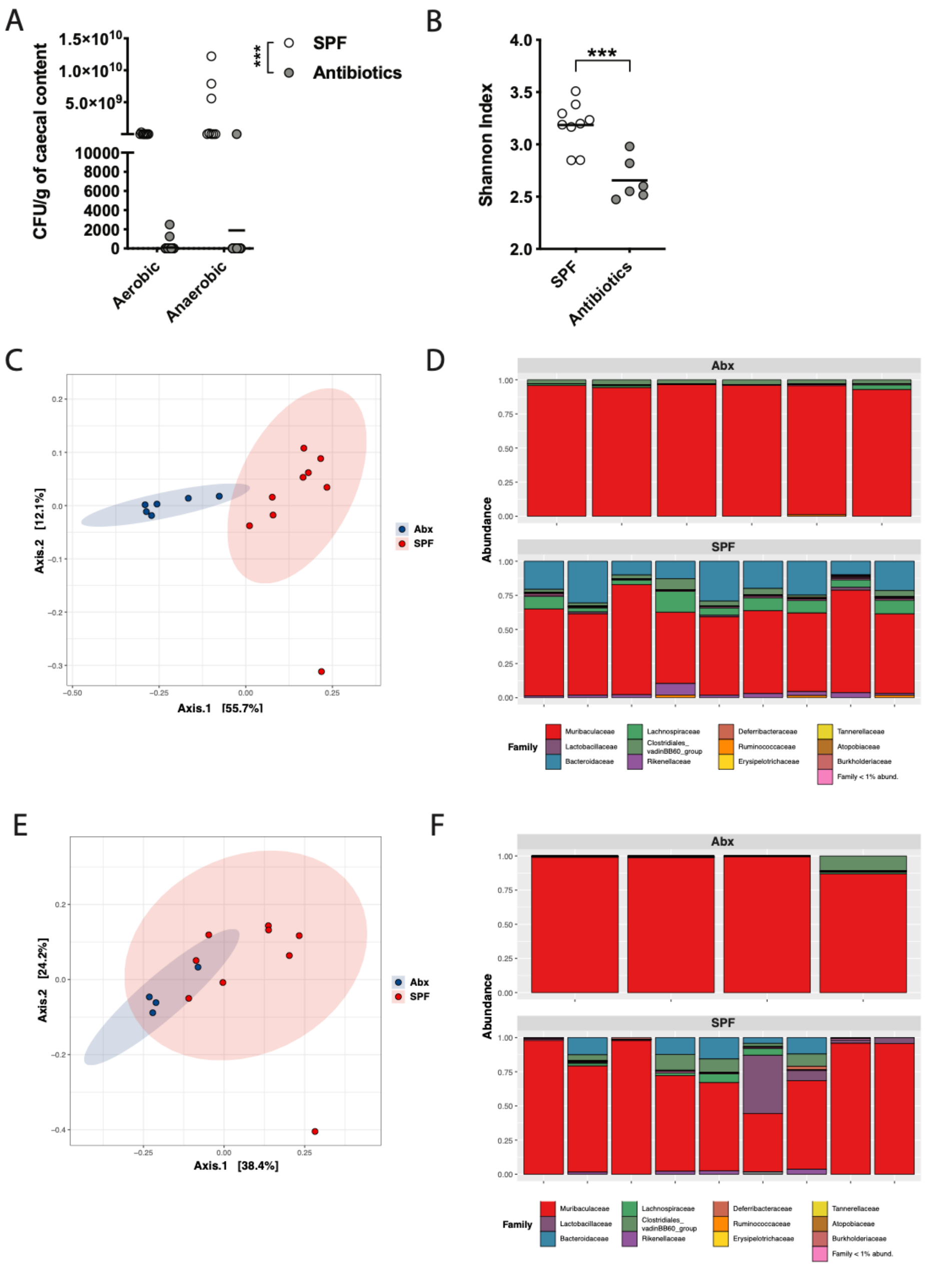


**Figure S1: Antibiotics treatment effectively reduces small intestinal microbial communities.**

Cecum and ileum content were collected from SPF and antibiotic treated mice (Enrofloxacin for 2 weeks and amoxicillin, acid clavulanic cocktail for the rest of the experiment). (A) Cecal content was cultured in either aerobic or anaerobic conditions to quantify live bacteria. Data were pooled from 5 independent experiments and were analysed using two-way ANOVA. The number of mice per group are: i) For SPF mice n= 8; ii) For antibiotic-treated mice n=37. The bacterial community in cecum (B-D) and ileum (E-F) was assessed using 16S rRNA amplicon sequencing (n=9 per group). (B) Shannon diversity index, (C and E) principal coordinates analysis (PCoA) using the Bray-Curtis dissimilarity based on amplicon sequence variants and (D and F) taxonomic classification on family level. Of note, 3/9 cecal and 5/9 ileal samples of antibiotic treated mice showed fewer than 500 reads and were excluded from analysis. All sequencing data sets are available in the NCBI BioProject database under accession number PRJNA799068.

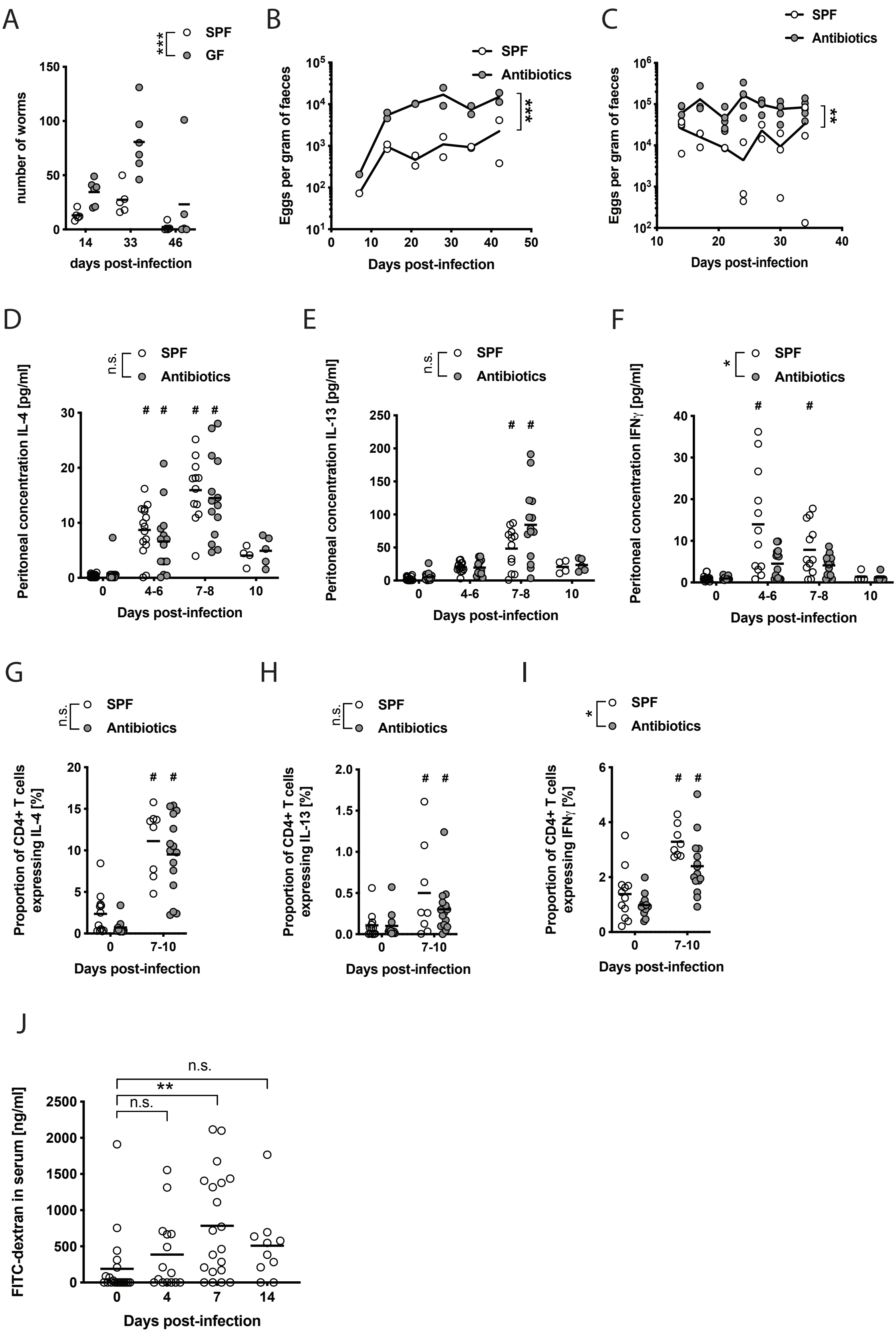


**Figure S2: Upon *H. polygyrus* infection local immune response is not dramatically affected in absence of microbiota**

(A) SPF or germ-free mice were infected for 14 to 46 days with 200 *Hpb* larvae. Number of adult worms in the intestine were assessed at each time points. Data are pooled from 2 independent experiments for day 14, one

experiment for day 33 and one experiment for day 46. The number of mice per time point are: i) for SPF mice D14=5, D33=5 and D46=5; ii) for germ-free mice D14=6, D33=6 and D46=5. (B and C) SPF and antibiotics treated mice (enrofloxacin for 2 weeks and amoxicillin, acid clavulanic cocktail for the rest of the experiment) were infected with 200 *Hpb* larvae. Number of eggs in the feces were assessed from (B) cages) or (C) individual animals in different experiments at shown time points and the data in (C) analysed using two way ANOVA. (B) Each dot represents a cage with 5 animals. (C) The number of mice per group are: i) For SPF mice n=3; ii) For antibiotic-treated mice n=5. (D to I) SPF and antibiotic-treated mice (enrofloxacin for 2 weeks then amoxicillin and clavulanic acid for the rest of the experiment) were infected for 0 to 10 days with 200 *Hpb* larvae. D) The number of mice per time point are: i) For SPF mice D0=19, D4-6=15, D7-8=12 and D10=4; ii) for antibiotic-treated mice D0=16, D4-6=14, D7-8=14 and D10=5. (E) The number of mice per time point are: i) For SPF mice D0=20, D4-6=14, D7-8=12 and D10=4; ii) for antibiotic-treated mice D0=18, D4-6=13, D7-8=14 and D10=5. (F) The number of mice per time point are: i) For SPF mice D0=19, D4-6=17, D7-8=19 and D10=13; ii) For antibiotic-treated mice D0=20, D4-6=18, D7-8=20 and D10=10.

(D, E, F) Blood was collected and concentration of (D) IL-4, (E) IL-13, (F) IFNγ in the serum were measured at each time points by ELISA. Data were pooled from 1 to 4 independent experiments and analysed using 2way ANOVA. (G, H, I) $CD4^+$ T lymphocytes were recovered from mesenteric lymph nodes and stimulated by PMA and ionomycin. Proportion of $CD4^+$ T cells producing (G) IL-4, (H) IL-13 and (I) IFNγ were measured using FACS. Data were pooled from 3 independent experiments with at least 12 individuals per group and were analysed using two-way ANOVA. (G, H, I) The number of mice per time point are: i) For SPF mice D0=12 and D7-10=8; ii) For antibiotic-treated mice D0=12 and D7-10=15. (J) SPF mice were infected for 0 to 14 days with 200 *Hpb* larvae. After 0, 4, 7 or 14 days of infection mice were orally gavaged with dextran-FITC. The presence of dextran-FITC in the blood was measured after one hour using fluorescence reader. Data were pooled from 2 to 4 independent experiments and analysed using Kruskal-Wallis. The number of mice per time point are: D0=20, D4=15, D7=20 and D14=10. * display statically significant differences between groups and # display statistically significant differences compared to the naïve group (0 days post infection).

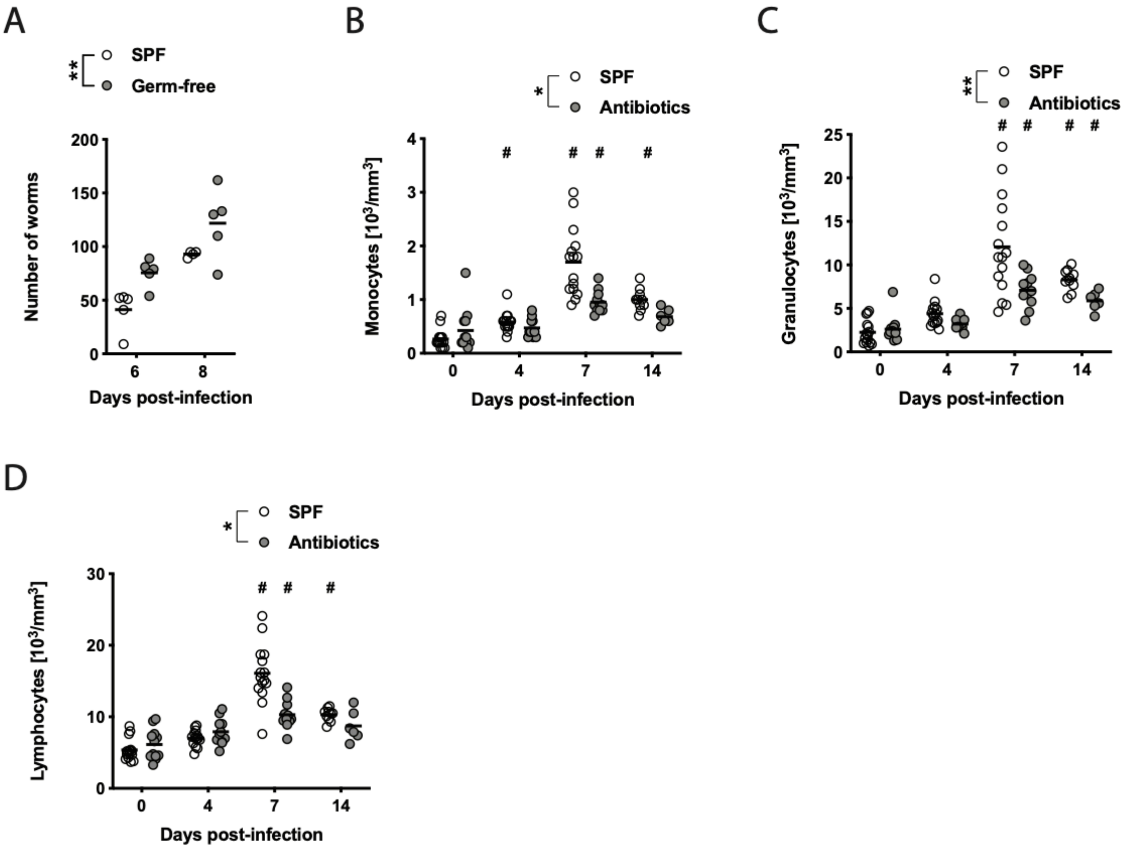


**Figure S3: Antibiotics treatment in *Hpb* infected mice reduced circulating white blood cells.**

(A) SPF or germ-free mice were infected for 6 and 8 days with 200 *Hpb* larvae. Number of larvae in the intestine were assessed at each time points. The number of mice per time point are: i) for SPF mice D6=4 and D8=4; ii) for germ-free mice D6=4 and D8=14. (B, C and D) SPF and antibiotic-treated mice (enrofloxacin for 2 weeks then amoxicillin and clavulanic acid for the rest of the experiment) were infected for 0 to 10 days with 200 *Hpb* larvae. Circulating (B) monocytes, (C) granulocytes and (D) lymphocytes were measured using a cell counter. Data are pooled from 2 to 3 independent experiment and analysed using two-way ANOVA. For (B, C, D) The number of mice per time point are: i) for SPF mice D0=15, D4=14, D7=15 and D14=10; ii) for antibiotic-treated mice D0=12, D4=11, D7=11 and D14=6 * display statically significant differences between groups and # display statistically significant differences compared to the naïve group (0 days post infection).

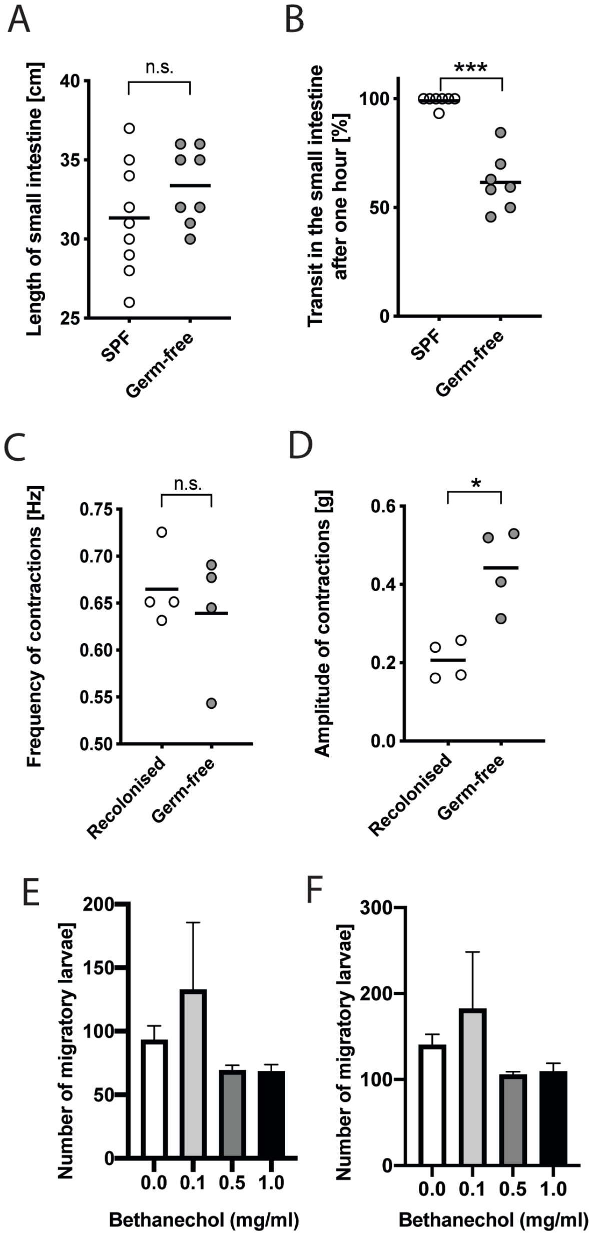


**Figure S4: Germ-free mice showed a reduced peristalsis and an increased amplitude of spontaneous intestinal muscle contractions.**

(A) The length of the small intestine from naive germ-free or recolonized germ-free mice was measured using a ruler. Data from two independent experiments were pooled and analysed using Mann-Whitney. The number of mice per group are: i) for SPF mice n=9; ii) for germ-free mice D6=8. (B) Transit was measured using carmine dye, the percentage of small intestinal length travelled one hour after gavage was reported. Data from two independent experiment were pooled and analysed using Mann-Whitney. The number of mice for each group is n=7. (C and D) Intestinal muscle contractions were analysed *ex vivo* using an organ bath and force transducers. Average (C) frequency and (D) amplitude were calculated from the contraction pattern recorded. Data are from one experiment and were analysed using Mann-Whitney. The number of mice for each group is n=4.* display statically significant differences between groups. (E and F) 200 *Hpb* L3 larvae were incubated with the indicated doses of bethanechol in saline at 37 °C for 10 minutes then larval migration assessed with a modified Baermann apparatus at 30 minutes and at 60 minutes. Data are from one experiment and were analysed using Mann-Whitney.

## Supplementary References:


1. Wypych TP, Pattaroni C, Perdijk O, Yap C, Trompette A, Anderson D *et al.* Microbial metabolism of L-tyrosine protects against allergic airway inflammation. *Nat Immunol* 2021; **22**(3)**:** 279-286.
